# Impact of Venoarterial Extracorporeal Membrane Oxygenation on Alkaline Phosphatase Metabolism after Cardiac Surgery

**DOI:** 10.3390/biom11050748

**Published:** 2021-05-17

**Authors:** Thomas Poschner, Anne-Kristin Schaefer, Doris Hutschala, Georg Goliasch, Julia Riebandt, Klaus Distelmaier, Martin H. Bernardi, Martin Andreas, Ruud Brands, Tandis Aref, Günther Laufer, Dominik Wiedemann

**Affiliations:** 1 Department of Cardiac Surgery, Medical University of Vienna, 1090 Vienna, Austria; thomas.poschner@meduniwien.ac.at (T.P.); anne-kristin.schaefer@meduniwien.ac.at (A.-K.S.); julia.riebandt@meduniwien.ac.at (J.R.); martin.andreas@meduniwien.ac.at (M.A.); tandis.aref@meduniwien.ac.at (T.A.); guenther.laufer@meduniwien.ac.at (G.L.); 2 Division of Cardiac Thoracic Vascular Anesthesia and Intensive Care Medicine, Medical University of Vienna, 1090 Vienna, Austria; doris.hutschala@meduniwien.ac.at (D.H.); martin.bernardi@meduniwien.ac.at (M.H.B.); 3 Department of Internal Medicine II, Division of Cardiology, Medical University of Vienna, 1090 Vienna, Austria; georg.goliasch@meduniwien.ac.at (G.G.); klaus.distelmaier@meduniwien.ac.at (K.D.); 4 Alloksys Life Sciences BV, 6708 PW Wageningen, The Netherlands; r.brands1@uu.nl; 5 Institute for Risk Assessment Sciences (IRAS), University of Utrecht, 3584 CM Utrecht, The Netherlands

**Keywords:** alkaline phosphatase, cardiac surgery, cardiopulmonary bypass, inflammation, SIRS, VA-ECMO, AKI

## Abstract

(1) Alkaline phosphatase (AP) is consumed during cardiopulmonary bypass (CPB). A high AP depletion leads to an impaired outcome after cardiac surgery. However, data is scarce on the postoperative course of AP under venoarterial ECMO (VA-ECMO) support. (2) A total of 239 patients with VA-ECMO support between 2000 and 2019 at the Department of Cardiac Surgery (Vienna General Hospital, Austria) were included in this retrospective analysis. Blood samples were collected at several timepoints (baseline, postoperative day (POD) 1–7, POD 14 and 30). Patients were categorized according to the relative AP drop (<60% vs. ≥60%) and ECMO duration (<5 days vs. ≥5 days). (3) Overall, 44.4% reached the baseline AP values within 5 days—this was only the case for 28.6% with a higher AP drop (compared to 62.7% with a lower drop; *p* = 0.000). A greater AP drop was associated with a significantly higher need for renal replacement therapy (40.9% vs. 61.9%; *p* = 0.002) and an impaired 1-year survival (51.4% vs. 66.0%; *p* = 0.031). (4) CPB exceeds the negative impact of VA-ECMO; still, ECMO seems to delay alkaline phosphatase recovery. A greater initial AP drop bears the risk of higher morbidity and mortality.

## 1. Introduction

The deleterious effects of cardiopulmonary bypass (CPB) are profound and involve various cellular levels—inter alia: endothelial hyperpermeability, ischemia–reperfusion injury, and direct endothelial cell injury [1,2]. Systemic inflammatory response syndrome (SIRS) virtually occurs in a third of patients after CPB and carries the potential for end-organ damage and increased morbidity [3]. Yet, this complex biochemical mechanism is not entirely understood. Systemic inflammation is triggered by a release of endogenous molecules. For example, adenosine triphosphate (ATP) is released from inflammatory or apoptotic cells [4] or from cells under duress—such as in the case of ischemic stress, where cell membranes experience temporal damage [5]. Moreover, alterations in flow patterns induce ischemic conditions within the gastrointestinal tract (GIT), resulting in an increased endothelial permeability with an impaired intestinal barrier function [6,7]. Thus, endotoxins (bacterial lipopolysaccharides (LPS)) of the gastrointestinal flora may enter the bloodstream [7]. LPS further promote an immunological response with the release of proinflammatory mediators—in particular, tumor necrosis factor (TNF)-alpha or interleukins (IL) such as IL-6 [8]. However, leaked ATP partly suppresses inflammation by stimulating CD39 and CD73 to produce anti-inflammatory adenosine, highlighting the complex interplay between the pro- and anti-inflammatory mediators [9].

Although alkaline phosphatase (AP) has been used for years in the diagnosis and/or observation of bone and liver diseases and is present almost ubiquitously in the human body [10,11], its role in immune response after surgery has only been recently investigated. Consequently, the complex physiological function of AP is not fully comprehended. The proinflammatory state triggered by cardiac surgery is counteracted on different cellular levels, including by serum alkaline phosphatase. AP neutralizes LPS and dephosphorylates extracellular adenosine diphosphate (ADP) or ATP to anti-inflammatory adenosine [4,12,13]. Recently, the phenomenon of AP consumption during cardiopulmonary bypass and the related adverse effects were demonstrated [14,15,16].

Postcardiac surgery extracorporeal membrane oxygenation (ECMO) is mainly considered in failed weaning from cardiopulmonary bypass, usually owing to respiratory or univentricular and/or biventricular failure [17]. ECMO represents no conclusive treatment but serves as a bridge to recovery, transplant, or durable mechanical circulatory support systems [18]. Compared to CPB, the immunologic effects owed to ECMO have been scarcely studied to date. To our knowledge, there is no study available highlighting the course of alkaline phosphatase on VA-ECMO support.

We hypothesize that ECMO support triggers systemic inflammation by blood contact with artificial surfaces and altered flow properties similar to full CPB, resulting in further alkaline phosphatase consumption and/or a delayed return to the baseline values. We want to investigate the course of alkaline phosphatase on post-cardiotomy VA-ECMO, which is dependent on the ECMO duration and the initial drop of alkaline phosphatase.

## 2. Materials and Methods

### 2.1. Patients

A total of 479 patients who required VA-ECMO support between 2000 and 2019 at the Department of Cardiac Surgery (Vienna General Hospital, Austria) were entered into the local ECMO registry. This clinical trial was submitted and approved by the local ethics committee of the Medical University of Vienna (1086/2019).

### 2.2. Laboratory Data

Alkaline phosphatase was determined as a routine parameter during admission and regularly in the postoperative course at our department. The reference range for alkaline phosphatase was between 40 and 130 U/L. Plasma concentrations were processed by means of enzyme kinetic measurement of native or heparinized samples at the department of Laboratory Medicine of the Medical University of Vienna. Blood samples were collected at the baseline and on each of the first seven postoperative days (POD), as well as on POD 14 (±1) and 30 (±3). The baseline was defined as a preoperative blood draw within a maximum of three weeks prior to surgery.

### 2.3. Follow-Up

Medical records were viewed to assess the individual postoperative course and the occurrence of adverse events. The local hospital’s patient documentation system, AKIM (SAP), was used for this purpose. Survival was determined with the help of federal statistics (Statistics Austria, Vienna, Austria).

### 2.4. Statistical Analysis

Categorical variables were expressed in numbers and percentages. Continuous variables were reported in mean ± standard deviation (SD) and median with the interquartile range (Q3, Q1), respectively, after testing for normal distribution (Kolmogorov–Smirnov test). Patients were stratified according to ECMO duration and initial AP drop (Equation (1)).

AP drop = 1 − AP POD 1/AP Baseline(1)

The cut-offs were defined using a receiver operating characteristics (ROC) analysis for 1-year mortality. Furthermore, we assessed the first day of when alkaline phosphatase returned or exceeded the baseline level. The nonparametric Mann–Whitney *U* Test and the Pearson’s chi-squared test or the Fisher’s Exact Test were used to assess the differences between the respective cohorts. The differences between AP Baseline and AP POD 1 and AP POD 30, respectively, were determined using the Wilcoxon signed-rank test. To reduce the likelihood of a type I error, Bonferroni correction was used to obtain an altered α for comparisons of the same dependent variables with a k of 2 (Equation (2)).

α_altered_ = 0.05/2 = 0.025 (2)

These findings are presented by means of boxplots. Survival was visualized with Kaplan–Meier curves. The differences between groups were tested using the log-rank test. All analyses were performed using SPSS version 27.0 (IBM Corp, Armonk, NY, USA). *p*-values were considered statistically significant, with the alpha level set at <0.05.

## 3. Results

### 3.1. Patient Population and Cut-Off Values

Two hundred and thirty-nine patients were included in the retrospective data analysis after reviewing their eligibility. The reasons for exclusion are listed in the Supplementary Data (Figure S1). The study cohort was divided according to the VA-ECMO support duration and the initial drop of alkaline phosphatase. The cut-off for the ECMO duration was set at five days, which was determined using a ROC analysis with an area under the curve (AUC) of 0.617 at a sensitivity of 57% and specificity of 61.3% for 1-year mortality. The factors, including the median ECMO duration and the results of previous studies, influenced this cut-off value. The sample size was 119 for the ECMO support of ≥5 days and 120 for the ECMO support of <5 days, respectively. Similarly, the patients were categorized according to the initial alkaline phosphatase drop. With an AUC of 0.614, the cut-off of an AP drop ≥ 60% revealed a sensitivity of 54% and a specificity of 62.5% for 1-year mortality. One hundred and five patients with an AP drop ≥ 60% and 110 patients with an AP drop < 60% were analyzed. Similarly, the decision for this cut-off was influenced by the previous study results using a 50% AP drop cut-off. If not stated otherwise, the second value refers to the longer ECMO duration (≥5 days) and the higher AP drop (≥60%).

### 3.2. Preoperative Characteristics

The patients’ baseline characteristics are illustrated in Table 1. The percentage of female patients revealed no significant differences between the different cohorts (overall: 38.5% | ECMO duration: 35.0% vs. 42.0%; *p* = 0.265 | AP Drop: 40.9% vs. 40.0%; *p* = 0.892). The patients with a higher AP drop were significantly older (64 (72, 55) vs. 70 (75, 62); *p* = 0.001) and had a significantly lower BMI (27.7 (30.8, 24.9) vs. 26.0 (30.4, 23.6); *p* = 0.045). Apart from a significantly higher portion of hypertension (66.7% vs. 81.7%; *p* = 0.012) and a significantly worse estimated glomerular filtration rate (eGFR) (63.7 (85.8, 45.3) vs. 55.5 (74.8, 38.4); *p* = 0.033) in patients with a higher alkaline phosphatase drop, no further significant differences were observed in the baseline characteristics.

### 3.3. Procedural Data

No differences in EuroScore II were observable between any cohorts (ECMO duration: 16.1 (36.5, 4.8) vs. 18.9 (32.7, 8.7); *p* = 0.320 | AP Drop: 16.0 (35.6, 5.6) vs. 18.6 (32.2, 8.9); *p* = 0.333). The higher alkaline phosphatase drop cohort revealed significantly longer procedural data—total surgery time (8.1 (9.9, 6.0) vs. 9.3 (12.1, 7.5) in h; *p* = 0.000), total cardiopulmonary bypass time (244 (326, 177) vs. 299 (411, 200) in min; *p* = 0.003), and total aortic cross clamp time (120 (169, 73) vs. 137 (215, 87) in min; *p* = 0.026)—as well as a significantly longer need for VA ECMO support (4.7 (6.7, 2.9) vs. 5.3 (9.1, 3.5) in days; *p* = 0.048). The procedural data is depicted in Table 2.

### 3.4. Alkaline Phosphatase

**Figure 1:**
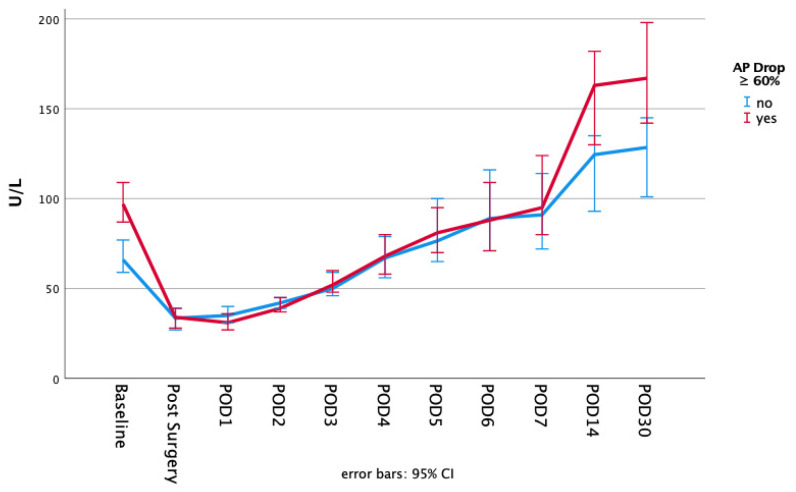
Alkaline phosphatase blood values dependent in the initial AP drop at the baseline on each consecutive day until POD 7, as well as on POD 14 and POD 30.

**Figure 2:**
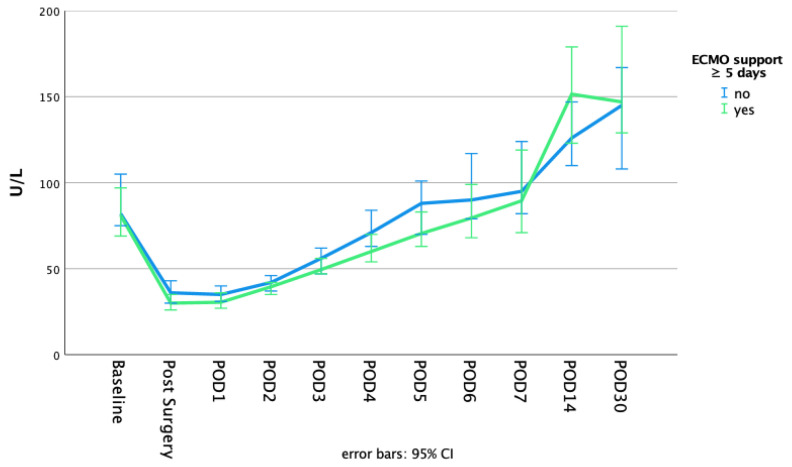
Alkaline phosphatase blood values dependent on the ECMO duration at the baseline on each consecutive day until POD 7, as well as on POD 14 and POD 30.

**Figure 3:**
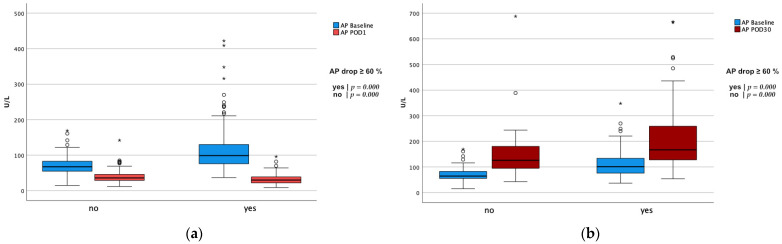
Comparison of the alkaline phosphatase levels at the baseline to different postoperative timepoints dependent on the initial drop of alkaline phosphatase. *p*-values were calculated for differences between the timepoints using the Wilcoxon signed-rank test. Statistical significance was obtained with an altered α level (set at 0.025). Using SPSS, values between the 1.5- and 3-fold IQR of Q3 are labeled as outliers and marked with a circle (°). Extreme outliers (3-fold IQR of Q3) are marked with an asterisk (*). (**a**) Comparison of AP baseline to POD 1. (**b**) Comparison of AP baseline to POD 30.

The postoperative course of alkaline phosphatase dependent on the initial AP drop and ECMO duration is highlighted in Figure 1 and Figure 2. The alkaline phosphatase levels at the baseline, as well as on POD 30, were significantly higher in the initial greater AP drop cohort (baseline: 68 (83, 55) vs. 99 (130, 76); *p* = 0.000 | POD30: 126 (185, 94) vs. 167 (262, 126); *p* = 0.001). The alkaline phosphatase blood levels on POD 30 compared to the baseline levels were significantly higher in both of the AP drop cohorts (*p* = 0.000 for both). The differences that were dependent on the initial AP drop between the baseline and POD 1 or POD 30, respectively, are presented in Figure 3. There were no significant differences between the short or long ECMO durations regarding the alkaline phosphatase levels at the baseline or on POD 30 (baseline: 81 (106, 61) vs. 80 (108, 60); *p* = 0.927; POD 30: 136 (198, 100) vs. 148 (221, 119); *p* = 0.133). However, comparing the baseline alkaline phosphatase levels to those on POD 30, values on POD 30 were significantly higher in both of the ECMO duration cohorts (*p* = 0.000 for both). Patients with an initially greater AP drop required more time to achieve the baseline values (baseline within POD5 and POD7: 62.7% vs. 28.6%; *p* = 0.000 | 80.9% vs. 49.5%; *p* = 0.000). This finding, however, was not observable in the cohorts based on the different ECMO support durations (baseline within POD 5 and POD7: 41.7% vs. 47.1%; *p* = 0.401 | 60.0% vs. 68.9%; *p* = 0.150). Therefore, 44.4% and 64.4% achieved the baseline values within POD5 and POD7 in the overall cohort. All of the laboratory values are provided in the Supplementary Data (Table S1).

### 3.5. Adverse Events and Mortality

The adverse events and outcome data are available in Table 3. Longer ECMO support was significantly associated with a higher incidence of adverse events, as well as a higher mortality. Bleeding complications, stroke, and the need for renal replacement therapy were significantly higher in patients who had longer extracorporeal membrane oxygenation (44.2% vs. 60.5%; *p* = 0.011 | 11.7% vs. 23.5%; *p* = 0.016 | 41.7% vs. 61.3%; *p* = 0.002). Similarly, these patients needed longer respiratory support (days until extubation/decannulation: 9 (15, 4) vs. 15 (26, 10); *p* = 0.000) and were more likely to die on the ECMO before extubation/decannulation on the ICU ward and in hospital (20.0% vs. 37.8%; *p* = 0.002 | 29.4% vs. 57.1%; *p* = 0.000 | 35.0% vs. 59.7%; *p* = 0.000 | 40.8% vs. 60.5%; *p* = 0.002). Likewise, the 30-day mortality, post-weaning mortality, and 1-year mortality were significantly higher in the longer ECMO support cohort (30.8% vs. 50.4%; *p* = 0.002 | 31.1% vs. 54.6%; *p* = 0.000 | 51.3% vs. 69.5%; *p* = 0.004).

A greater initial AP drop was associated with a higher incidence of adverse events. Patients with an alkaline phosphatase drop greater than 60% were significantly more likely to experience bleeding complications (40.0% vs. 60.0%; *p* = 0.003) and required renal replacement therapy (40.9% vs. 61.9%; *p* = 0.002). Similarly, a significantly higher number needed tracheotomy (20.9% vs. 35.2%; *p* = 0.019). There was a strong trend towards higher ICU mortality (38.2% vs. 51.4%; *p* = 0.051) and a significantly worse outcome after one year (51.4% vs. 66.0%; *p* = 0.031).

**Figure 4:**
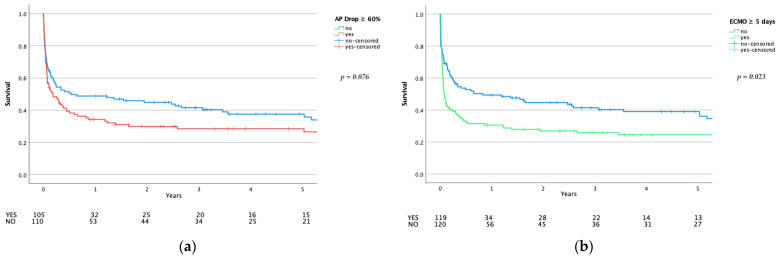
Five-year survival. (**a**) Long-term survival comparison dependent on the initial drop of alkaline phosphatase. (**b**) Long-term survival comparison dependent on the ECMO duration. *p*-values calculated using the log-rank test.

Long-term survival (5-year) was significantly worse after long ECMO support (*p* = 0.023) and showed at least a negative trend after an initial greater drop in alkaline phosphatase (*p* = 0.076)—see Figure 4.

## 4. Discussion

Consistent with the literature, longer ECMO support is associated with a worse outcome in this study [19]. However, not solely the length of ECMO support but, also, the consumption of alkaline phosphatase during cardiac surgery seems to have an essential role in postoperative recovery. The greater the drop, the higher the morbidity and mortality. Extended cardiopulmonary bypass times have a significant effect on increased alkaline phosphatase consumption. This increased alkaline phosphatase consumption is reflected in a higher necessity for longer ECMO duration—on average, half a day. This highlights the importance of counteracting the initial trauma caused by the deleterious effects of extracorporeal circulation to protect patients from prolonged (and/or general) ECMO usage.

**Figure 5:**
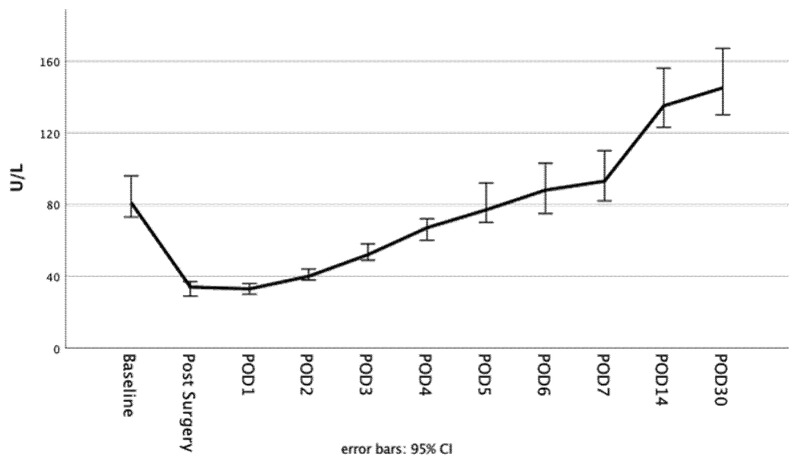
Postoperative course of alkaline phosphatase in the overall cohort until POD 30.

Surgical trauma, however, appears to be of such great magnitude that the foreign surface and the altered flow conditions during extracorporeal membrane oxygenation only have a minor impact on the further course of alkaline phosphatase—apparent in reaching the nadir of alkaline phosphatase depletion already on POD 1 across all cohorts. The duration of ECMO has no significant impact on the speed of the alkaline phosphatase recovery, indicated in the marginal differences observed in time to achieve the baseline values (the percentage of patients who achieved the baseline levels within POD 5 and POD 7: 41.7% vs. 47.1%; *p* = 0.401 | 60.0% vs. 68.9%; *p* = 0.150). However, a possible prolonged recovery to the baseline values is evident compared to patients without ECMO support [14,20]. While Schaefer et al. [14] described a timespan of nearly 3 to 4 days to reach close to the baseline values (without giving a precise definition), the present study highlighted a period of 5 days in the overall cohort (see Figure 5). However, in patients affected by an initial greater drop, recovery is significantly delayed (percentage of patients who achieved the baseline levels within POD 5 and POD 7: 62.7% vs. 28.6%; *p* = 0.000 | 80.9% vs. 49.5%; *p* = 0.000). Nevertheless, the comparison between the current study and the trial by Schaefer et al. was limited, as a relatively high portion with higher AP drop required VA-ECMO, and different cut-off values were chosen. Further prospective studies are necessary to investigate this effect.

With the exception of one, there was no significant difference regarding the index surgical procedure between the cohorts (Supplementary Data Table S2). Only the number of patients who underwent concomitant CABG and valve surgery was significantly higher in the greater AP drop cohort (24.5% vs. 38.1%; *p* = 0.032). While the total surgery time was significantly longer in these patients (9.17 (10.00, 6.92) vs. 9.63 (12.13, 8.02) in hours; *p* = 0.035), there was no statistically significant difference between CPB and ACC times (*p* = 0.218 and *p* = 0.312, respectively). This might highlight the occurrence of intraoperative complications, such as bleeding events and/or difficulties in preparation and cannulation before and after CPB. Besides, the postoperative morbidity in these patients was significantly elevated with a higher rate of any bleeding complications (29.6% vs. 72.5%; *p* = 0.001). We cannot disclose the exact causative reason for the greater portion in the higher AP drop cohort owing to the retrospective nature of this study.

In excluding almost 50% of the patients of the local ECMO registry, we aimed to provide a homogenous study cohort, accepting a probable selection bias (for the exclusion criteria, see Supplementary Data Figure S1). Furthermore, we intended to analyze only the patients with a similar rationale for VA-ECMO implantation, this being the underlying reason for excluding most of the patients (124/479). The exclusion of ECMO reimplantation and short ECMO duration had several considerations: (1) to minimize further confounders of the postoperative AP course, (2) the minor impact of VA-ECMO expected owing to the short duration, and (3) no sufficient conclusion possible based on the low number of patients. Further prospective studies are needed to analyze the course of AP in different settings.

Although the medical complexity of ECMO patients can hardly be simplified to a single parameter, we were able to identify one surrogate (alkaline phosphatase) apparently having a significant impact on morbidity and mortality, highlighting the central role in combating proinflammatory agents after cardiac surgery. Nevertheless, AP alone was insufficient to fulfill the role as a risk assessment tool due to its poor predictive power (AUC of 0.614). A sensitivity and specificity of 54% and 62.5%, respectively, for a 60% drop cut-off of alkaline phosphatase were only indicative rather than of a valid predictive nature. Different cut-off values with their respective sensitivity and specificity are provided in the Supplementary Data (Table S3). Alterations of the physiological conditions and an iatrogenic trauma result in increased levels of endotoxins and other proinflammatory mediators [2,7]. Extracellular ATP promotes NLRP3, an inflammasome complex, that enhances cytokine production and tissue injury in acute kidney injury (AKI) [21]. Alkaline phosphatase, however, dephosphorylates ATP into reno-protective adenosine [22]. This combat of proinflammatory mediators decreases the alkaline phosphatase concentration in blood [15,23]. Once, or if proinflammatory mediators are abundant, SIRS may occur. The increased need for renal replacement therapy after an initial greater drop of alkaline phosphatase in our cohort is of major importance, as patients suffering from AKI are generally prone to an independently worse outcome [24]. While the baseline creatinine levels were not statistically different between the cohorts, the patients with a higher AP drop revealed a significantly worse baseline renal clearance (*p* = 0.033). The median values of both AP drop cohorts were compatible with an impaired kidney function of mild classification or above. The clinical impact, however, is most likely of minor relevance, considering a median net difference of only 8 mL/min. Thus, the detrimental outcome in terms of the necessity for dialysis may be related to the negative effect of CPB and VA-ECMO rather than the baseline kidney function. Nevertheless, we cannot exclude a potential higher consumption of AP owed to impaired baseline renal function. In addition, the baseline pharmacological therapy was not analyzed in this study cohort. Thus, a potential interaction with AP cannot be excluded. The influence of baseline therapy on the postoperative course of AP compared to the extent of surgical intervention is questionable—although not excludable by this study.

Interestingly, alkaline phosphatase levels are consistently higher at 30 days compared to baseline. This effect is even more accentuated for patients affected by an initial greater AP drop. Cleavage of alkaline phosphatase from cell membranes in an excessive proinflammatory state renders endothelia vulnerable to hyperpermeability [20]. Potentially, such condition may serve as a trigger for increased endogenous alkaline phosphatases production to enhance the systems’ resistance to future trauma. Similar effects are described for a reduced myocardial injury after ischemic preconditioning [25]. Thereby, nuclear factor E2-related factor 2 (Nrf2) bonds with an antioxidant response element in the nucleus, increasing the cells’ resistance to oxidative stress [26]. However, this is of mere speculative nature and the causal relationship cannot be confirmed by this study. The clinical effect, the sustainability, and the possible adverse effects of elevated alkaline phosphatase levels require further investigation considering that long-term elevated alkaline phosphatase levels are associated with a promotion of vascular calcification [27].

External alkaline phosphatase substitution is the focus of current research [28]. Recent studies have shown improved renal function in sepsis patients [29]. An ongoing Phase III study (APPIRED III—ClinicalTrials.gov NCT03050476) is designed to evaluate the benefit of substituted alkaline phosphatase during cardiac surgery and supplied bovine alkaline phosphatase is expected to minimize the incidence of acute kidney injury with associated adverse events. While these trials only investigate the initial administration over 24 h, a continued substitution over the postoperative course might pose a clinical importance. A beneficial effect may be especially conceivable in the setting of VA-ECMO, as these patients are particularly susceptible for renal replacement therapy (51.5% in our study cohort).

## 5. Conclusions

The main finding of the study indicates a significantly higher morbidity and an impaired outcome after a higher drop of alkaline phosphatase at one year, as well as a negative trend in ICU and long-term survival. The negative impact on alkaline phosphatase metabolism of surgical trauma and CPB exceeds the impact of VA-ECMO.

## 6. Limitations

All limitations for a single center, retrospective study design apply for this study. Given the retrospective nature of this study and the long study period, many potential co-founders regarding outcome (increased center expertise and change of patient management, as well as the usage of different ECMO devices) were not taken into account. All assumptions apply only to post-cardiotomy VA-ECMO patients. The fundamental limitation of this study was the poor predictive value of alkaline phosphatase with an AUC of 0.614. Moreover, this trial lacks data on the baseline pharmacological management which presumably interferes as a confounder, as information was not available. Furthermore, we cannot make any assumptions on the relationship between laboratory chemical changes and patient management. A possible confounder—namely, the possibility or the extent of a greater AP drop due to the dilution effect—cannot be answered by this study. Still, an initial alkaline phosphatase consumption seems to have an impact on morbidity and mortality. The complexity of the patient’s health condition is difficult to represent in categorical and metric variables; therefore, patient management requires an individual, multidisciplinary decision rather than reliance on a specific set of laboratory parameters alone.

## Figures and Tables

**Table 1:** Preoperative data.

Baseline Specifics	Overall *n* = 239	ECMO < 5 Days *n* = 120	ECMO ≥ 5 Days *n* = 119	*p*-Value ª	AP Drop < 60% *n* = 110	AP Drop ≥ 60% *n* = 105	*p*-Value ª
age	68 (75, 58)	69 (77, 61)	67 (74, 57)	0.228	64 (72, 55)	70 (75, 62)	0.001 *
female	92 (38.5)	42 (35.0)	50 (42.0)	0.265	45 (40.9)	42 (40.0)	0.892
BMI	27.1 (30.8, 24.2)	26.7 (30.8, 24.5)	27.2 (30.4, 23.9)	0.884	27.7 (30.8, 24.9)	26.0 (30.4, 23.6)	0.045 *
hypertension	174 (73.7)	93 (78.8)	81 (68.6)	0.076	72 (66.7)	85 (81.7)	0.012 *
IDDM	22 (9.4)	9 (7.8)	13 (10.9)	0.405	12 (11.0)	9 (8.6)	0.580
preoperative dialysis	20 (8.4)	9 (7.5)	11 (9.2)	0.626	8 (7.3)	8 (7.6)	0.923
CVA	46 (19.2)	21 (17.5)	25 (21.0)	0.492	22 (20.0)	20 (19.0)	0.860
CAD	148 (61.9)	79 (65.8)	69 (58.0)	0.211	61 (55.5)	71 (67.6)	0.067
PCI	39 (16.3)	23 (19.2)	16 (13.4)	0.231	17 (15.5)	17 (16.2)	0.882
CABG	28 (11.7)	18 (15.0)	10 (8.4)	0.113	10 (9.1)	11 (10.5)	0.732
MI	85 (35.6)	46 (38.3)	39 (32.8)	0.369	40 (36.4)	37 (35.2)	0.863
CPR	19 (7.9)	7 (5.8)	12 (10.1)	0.225	10 (9.1)	8 (7.6)	0.697
NYHA III + IV	188 (79.7)	88 (75.2)	100 (84.0)	0.092	85 (80.0)	84 (80.8)	0.615
LVEF	45 (60, 30)	45 (60, 30)	45 (60, 25)	0.453	45 (60, 30)	45 (60, 29)	0.788

All values are referred to as the median (Q3, Q1) or total number (*n*) and percentage (%) | ª If not stated otherwise, the Mann–Whitney *U* Test and Pearson’s chi-squared test, respectively, were used; values marked with an asterisk (*) achieved statistical significance | Baseline characteristics for hypertension and IDDM were not available for 3 patients; NYHA classification was not evaluated in 4 patients. BMI—body mass index (kg/m^2^); IDDM—insulin dependent diabetes mellitus; CVA—cerebrovascular accident (including stroke and transient ischemia attacks in the patient’s history); CAD—coronary artery disease; PCI—percutaneous coronary intervention; CABG—coronary artery bypass grafting; MI—myocardial infarction; CPR—cardiopulmonary resuscitation; NYHA—New York Heart Association classification; LVEF—left ventricular ejection fraction (%).

**Table 2:** Procedural data.

Surgical Specifics	Overall *n* = 239	ECMO < 5 Days *n* = 120	ECMO ≥ 5 Days *n* = 119	*p*-Value ª	AP Drop < 60% *n* = 110	AP Drop ≥ 60% *n* = 105	*p*-Value ª
EuroScore II	18.2 (34.5, 7.5)	16.1 (36.5, 4.8)	18.9 (32.7, 8.7)	0.320	16.0 (35.6, 5.6)	18.6 (32.2, 8.9)	0.333
critical preoperative state	104 (43.5)	53 (44.2)	51 (42.9)	0.838	53 (48.2)	40 (38.1)	0.136
h/o cardiac surgery	73 (30.5)	34 (28.3)	39 (32.8)	0.456	31 (28.2)	30 (28.6)	0.949
active endocarditis	32 (13.4)	19 (15.8)	13 (10.9)	0.265	16 (14.5)	12 (11.4)	0.497
elective operation	109 (45.6)	54 (45.0)	55 (46.2)	0.850	46 (41.8)	53 (50.5)	0.203
urgent operation	58 (24.3)	26 (21.7)	32 (26.9)	0.346	28 (25.5)	25 (23.8)	0.780
emergency operation	68 (28.5)	38 (31.7)	30 (25.2)	0.269	34 (30.9)	25 (23.8)	0.244
salvage operation	4 (1.7)	2 (1.7)	2 (1.7)	1.000 º	2 (1.8)	2 (1.9)	1.000 º
surgery time	8.5 (10.4, 6.8)	8.5 (10.3, 6.4)	8.5 (10.7, 7.2)	0.341	8.1 (9.9, 6.0)	9.3 (12.1, 7.5)	0.000 *
CPB	258 (347, 185)	251 (337, 178)	276 (373, 196)	0.087	244 (326, 177)	299 (411, 200)	0.003 *
ACC	128 (181, 81)	122 (174, 78)	132 (194, 85)	0.322	120 (169, 73)	137 (215, 87)	0.026 *
VA-ECMO duration	4.9 (8.1, 3.1)	3.1 (3.9, 2.2)	8.1 (11.7, 5.9)	0.000 *	4.7 (6.7, 2.9)	5.3 (9.1, 3.5)	0.048 *

All values are referred to as the median (Q3, Q1) or total number (*n*) and percentage (%) | ª If not stated otherwise, the Mann–Whitney *U* Test and Pearson’s chi-squared test, respectively, were used; º *p*-values were calculated by Fisher’s Exact Test; values marked with an asterisk (*) achieved statistical significance | EuroScore II in %; Surgery time in hours; CPB—cardiopulmonary bypass in minutes; ACC—aortic cross clamp time in minutes; VA-ECMO duration in days.

**Table 3:** Adverse events and mortality.

Outcome	Overall *n* = 239	ECMO < 5 Days *n* = 120	ECMO ≥ 5 Days *n* = 119	*p*-Value ª	AP Drop < 60% *n* = 110	AP Drop ≥ 60% *n* = 105	*p*-Value ª
any stroke	42 (17.6)	14 (11.7)	28 (23.5)	0.016 *	20 (18.2)	17 (16.2)	0.699
any bleeding complication	125 (52.3)	53 (44.2)	72 (60.5)	0.011 *	44 (40.0)	63 (60.0)	0.003 *
need for any renal replacement therapy	123 (51.5)	50 (41.7)	73 (61.3)	0.002 *	45 (40.9)	65 (61.9)	0.002 *
days until first extubation/decannulation	13 (22, 7)	9 (15, 4)	15 (26, 10)	0.000 *	12 (20, 7)	14 (26, 7)	0.108
need for tracheotomy	62 (25.9)	29 (24.2)	33 (27.7)	0.530	23 (20.9)	37 (35.2)	0.019 *
Mortality …							
… on ECMO support	69 (28.9)	24 (20.0)	45 (37.8)	0.002 *	28 (25.5)	30 (28.6)	0.607
… within 30 days	97 (40.6)	37 (30.8)	60 (50.4)	0.002 *	36 (32.7)	45 (42.9)	0.125
… before extubation/decannulation	103 (43.3)	35 (29.4)	68 (57.1)	0.000 *	39 (35.5)	48 (46.2)	0.111
… post weaning	102 (42.9)	37 (31.1)	65 (54.6)	0.000 *	39 (35.5)	47 (45.2)	0.146
… on ICU ward	113 (47.3)	42 (35.0)	71 (59.7)	0.000 *	42 (38.2)	54 (51.4)	0.051
… in hospital	121 (50.6)	49 (40.8)	72 (60.5)	0.002 *	47 (42.7)	57 (54.3)	0.090
… 1 year	142 (60.4)	60 (51.3)	82 (69.5)	0.004 *	56 (51.4)	68 (66.0)	0.031*

All values are referred to as the median (Q3, Q1) or total number (*n*) and percentage (%) | ª If not stated otherwise, the Mann–Whitney *U* Test and Pearson’s chi-squared test, respectively, were used; values marked with an asterisk (*) achieved statistical significance | post-weaning mortality defined as death within 30 days after VA-ECMO explantation | Follow-up (FUP) for one patient was too short to assess ‘time until extubation/decannulation’, as well as mortality ‘before extubation/decannulation’, ‘post-weaning’, and ‘1 year’; ‘1-year mortality’ was not assessable for a total of 4 patients.

## Supplementary Materials

The following are available online at https://www.mdpi.com/article/10.3390/biom11050748/s1: Figure S1: Flow chart for patient selection; Figure S2: ROC analysis for AP drop and 1-year mortality; Table S1: Supplementary data for the laboratory parameters; Table S2: Supplementary data of the index procedure; Table S3: Sensitivity and Specificity of different AP drops values according to the AUC analysis. The cut-off of 60% demonstrated a balanced relation between sensitivity and specificity.
