# Supplementary material for: Impact of Venoarterial Extracorporeal Membrane Oxygenation on Alkaline Phosphatase Metabolism after Cardiac Surgery
Source: Biomolecules. 2021 May 17;11(5):748. doi: 10.3390/biom11050748 (PMC8156119; doi:10.3390/biom11050748)
Supplement: Supplementary file 1 [file biomolecules-11-00748-s001.zip › biomolecules-1194384-supplementary.pdf]

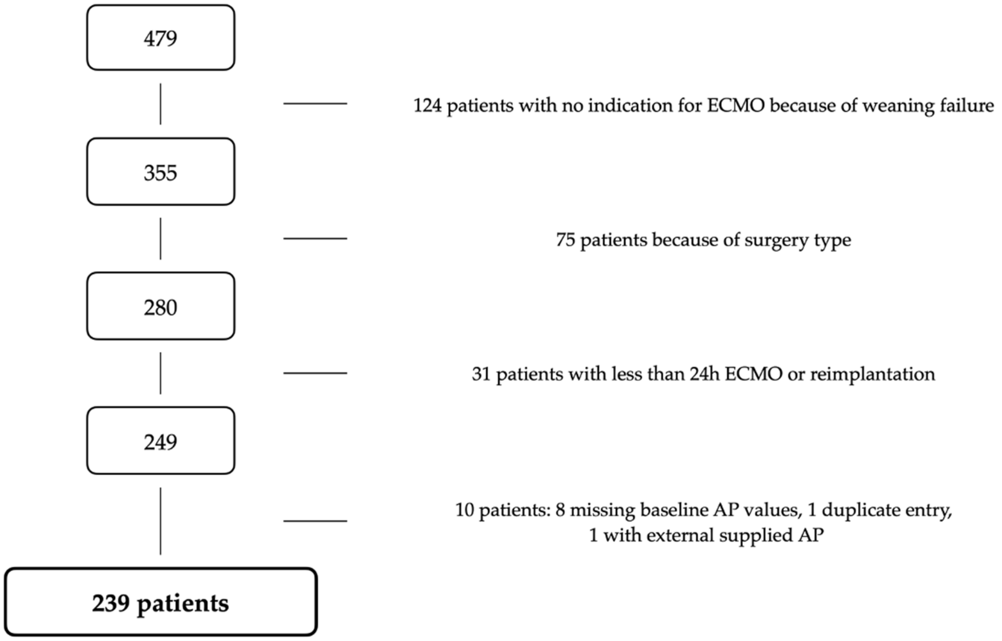

Figure S1. Flow chart for patient selection. For analysis purposes a total of 479 patients were screened from the local ECMO registry. 124 patients were excluded due to a VA-ECMO indication other than CPB weaning failure. These patients received VA-ECMO during the postoperative course because of hemodynamic decline, cardiopulmonary resuscitation, and/or respiratory failure after cardiac surgery within 30 days after surgery. 75 patients were excluded because of surgery - 61 patients who receiving a heart transplant and 14 patients with an unspecified intervention, ranging from a combined heart-lung transplantation, aortic annulus rupture after transcatheter aortic valve implantation, mediastinal cyst removal to ischemic ventricular septal defect. 16 patients who required VA-ECMO support for less than 24h and 15 patients who were in need of VA-ECMO re-implantation were excluded. A further 8 patients with missing baseline AP levels were excluded. One patient who participated at a study involving bovine alkaline phosphatase submission was excluded. One duplicate entry was excluded. Two-hundred-thirty-nine (239) patients entered the retrospective data analysis after reviewing the exclusion criteria.

Table S1. Supplementary data for the laboratory parameters.

	Overall<br>*n*=239	ECMO<br>< 5days<br>*n*=120	ECMO<br>≥ 5 days<br>*n*=119	*p*-value[a]	AP drop<br>< 60%<br>*n*=110	AP drop<br>≥ 60%<br>*n*=105	*p*-value[a]
Baseline AP within POD 5	106 (44.4)	50 (41.7)	56 (47.1)	0.401	69 (62.7)	30 (28.6)	0.000*
Baseline AP within POD 7	154 (64.4)	72 (60.0)	82 (68.9)	0.150	89 (80.9)	52 (49.5)	0.000*
Baseline AP within POD 30	193 (80.8)	92 (76.7)	101 (84.9)	0.108	96 (87.3)	81 (77.1)	0.052
Death before Baseline AP achieved	39 (16.3)	23 (19.2)	16 (13.4)	0.231	12 (10.9)	19 (18.1)	0.134
Drop of AP (POD1)	0.59 (0.69, 0.46)	0.56 (0.68, 0.46)	0.61 (0.69, 0.49)	0.166	0.47 (0.54, 0.40)	0.69 (0.75, 0.65)	0.000*
AP baseline	80 (107, 61)	81 (106, 61)	80 (108, 60)	0.927	68 (83, 55)	99 (130, 76)	0.000*
CRP baseline	1.0 (3.9, 0.3)	1.0 (3.8, 0.3)	1.1 (4.0, 0.3)	0.821	1.0 (4.8, 0.3)	0.9 (2.9, 0.4)	0.582
Creatinine level baseline	1.23 (1.68, 1.00)	1.23 (1.61, 1.02)	1.23 (1.75, 0.98)	0.958	1.20 (1.64, 0.97)	1.22 (1.69, 1.00)	0.589
eGFR	60.1 (83.5, 41.8)	60.1 (83.0, 40.0)	60.1 (83.5, 41.9)	0.922	63.7 (85.8, 45.3)	55.5 (74.8, 38.4)	0.033*
AP POD 1	34 (44, 27)	34 (44, 28)	33 (41, 26)	0.306	36 (46, 29)	30 (40, 22)	0.000*
CRP POD 1	5.5 (8.7, 3.6)	6.0 (9.5, 3.7)	5.3 (7.9, 3.4)	0.118	6.8 (10.5, 4.4)	4.9 (7.4, 3.4)	0.000*
Creatinine level POD 1	1.33 (1.73, 1.02)	1.35 (1.71, 1.00)	1.32 (1.75, 1.04)	0.921	1.29 (1.68, 0.98)	1.31 (1.65, 1.01)	0.952
AP POD 5	77 (112, 62)	82 (109, 65)	76 (116, 58)	0.319	77 (109, 64)	81 (119, 59)	0.610
CRP POD 5	14.7 (22.9, 8.0)	15.7 (23.8, 8.4)	14.3 (22.6, 7.8)	0.667	17.2 (23.5, 8.8)	12.5 (22.6, 7.2)	0.110
Creatinine level POD 5	1.41 (1.95, 1.03)	1.36 (1.85, 1.01)	1.53 (2.10, 1.07)	0.137	1.36 (1.91, 1.02)	1.43 (1.94, 1.05)	0.507
AP POD 7	103 (151, 74)	97 (150, 77)	105 (155, 72)	0.892	100 (149, 71)	102 (158, 75)	0.676
CRP POD 7	13.5 (19.1, 8.2)	12.7 (17.1, 8.2)	14.8 (20.4, 7.7)	0.330	13.9 (19.3, 8.1)	13.5 (19.3, 8.7)	0.491
Creatinine level POD 7	1.39 (1.91, 1.00)	1.31 (1.76, 0.99)	1.49 (2.03, 1.00)	0.205	1.20 (1.76, 0.95)	1.48 (1.89, 1.02)	0.243
AP POD 30	143 (211, 102)	136 (198, 100)	148 (221, 119)	0.133	126 (185, 94)	167 (262, 126)	0.001*
CRP POD 30	4.8 (9.3, 2.4)	4.2 (9.3, 2.4)	5.3 (9.5, 2.5)	0.770	4.2 (7.3, 2.5)	5.5 (10.0, 2.4)	0.362
Creatinine level POD 30	1.01 (1.54, 0.70)	1.02 (1.47, 0.70)	1.00 (1.83, 0.69)	0.894	0.99 (1.63, 0.68)	1.00 (1.43, 0.70)	0.829

All values are referred in median (Q3, Q1) or in total number (*n*) and percentage (%) ǀ [a] If not stated otherwise, Mann-Whitney-U-Test and Pearson's chi-squared test, respectively, were used; values marked with an asterisk (*) achieved statistical significanceǀ Reference range for alkaline phosphatase 40-130 U/L, CRP < 0.5 mg/dL and creatinine 0.7 – 1.2 mg/dL ǀ eGFR = estimated glomerular filtration rate calculated by the Cockroft-Gault formula.

Table S2. Supplementary data of the index procedure.

	**Overall** *n*=239	**ECMO** < 5days *n*=120	**ECMO** ≥ 5 days *n*=119	***p*-value**[a]	**AP drop** < 60% *n*=110	**AP drop** ≥ 60% *n*=105	***p*-value**[a]
Index procedure							
… CABG	34 (14.2)	19 (15.8)	15 (12.6)	0.475	18 (16.4)	11 (10.5)	0.207
… CABG + Valve Surgery	72 (30.1)	36 (30.0)	36 (30.3)	0.966	27 (24.5)	40 (38.1)	0.032*
… Valve Surgery	74 (31.0)	37 (30.8)	37 (31.1)	0.965	33 (30.0)	35 (33.3)	0.599
… Aortic Aneurysm	9 (3.8)	4 (3.3)	5 (4.2)	0.749º	6 (5.5)	1 (1.0)	0.120º
… Aortic Dissection	21 (8.8)	7 (5.8)	14 (11.8)	0.105	11 (10.0)	8 (7.6)	0.539
… Congenital	3 (1.3)	1 (0.8)	2 (1.7)	0.622º	2 (1.8)	1 (1.0)	1.000º
… Endocarditis	26 (10.9)	16 (13.3)	10 (8.4)	0.221	13 (11.8)	9 (8.6)	0.432

All values are referred in total number (*n*) and percentage (%) | [a] *p*-values calculcated with Pearson's chi-squared test if not stated otherwise; values marked with an asterisk (*) achieved statistical significance | º *p* values calculated by Fisher Exact Test | CABG – coronary aortic bypass grafting.

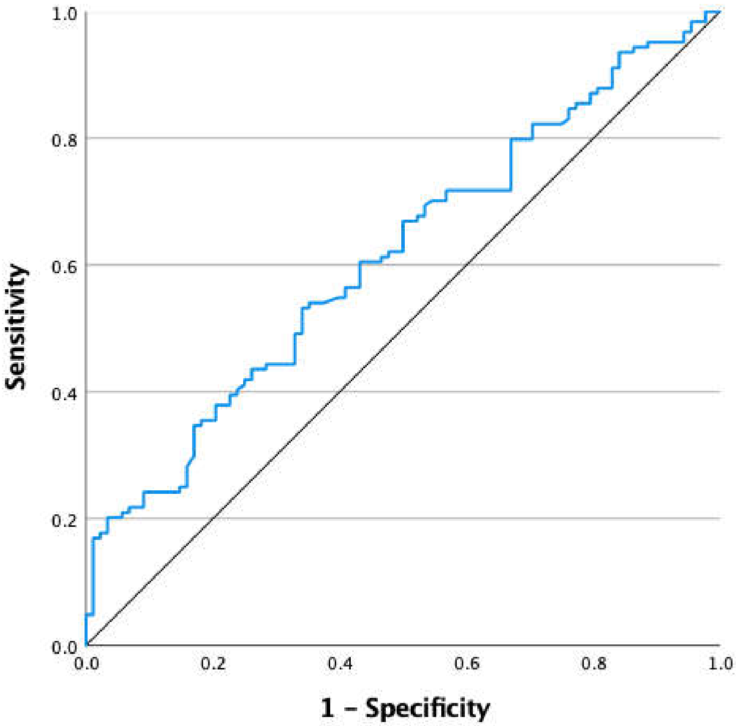

Figure S2. ROC analysis for AP drop and 1-year mortality.

Table S3. Sensitivity and Specificity of different AP drops values according to the AUC analysis. The cut-off of 60% demonstrated a balanced relation between sensitivity and specificity.

AP drop …	Sensitivity	Specificity
30%	95.2%	8%
40%	88.7%	17.0%
50%	71.8%	36.4%
**60%**	**54.0%**	**62.5%**
70%	25.0%	85.2%
80%	8.1%	98.9%
